# Optimal determination of Physician Global Assessment of lupus disease activity: a pilot study

**DOI:** 10.1186/ar4658

**Published:** 2014-09-18

**Authors:** Cynthia Aranow

**Affiliations:** 1 Feinstein Institute for Medical Research, Manhasset, NY, USA

## Background

The Physician Global Assessment (PGA) is an important and useful outcome measurement of lupus disease activity. Consensus on when the PGA should be performed is lacking; many lupus specialists determine the PGA at the time of a patient visit while others determine the PGA after receipt of laboratory values. The objective of this study was to collect preliminary data to determine the optimal time to perform a PGA.

## Methods

In this pilot study, a PGA was performed by a single clinician upon completion of an outpatient clinical encounter and again after receipt of pertinent laboratory values. The pre-laboratory PGA was determined with knowledge of the subject's previous laboratory values and lupus history, while the post-laboratory PGA was determined after laboratory reports from the patient clinical encounter were also available. Disease activity could range from 0 to 3, with 0 representing no disease activity, 1 representing mild disease activity, 2 representing moderate disease activity and 3 representing the most severe lupus disease imaginable. Laboratory values obtained at each clinical visit included a CBC, comprehensive chemistries, C3, C4, anti-dsDNA, urinalysis and, if pertinent, a spot urinary protein/creatinine ratio. Disease duration and SELENA SLEDAI were recorded. Results are presented using descriptive statistics; the paired Student's *t *test was utilized to compare the pre-laboratory PGA with the post-laboratory PGA.

## Results

Thirty-three patients, three male and 30 female with an average SLE disease duration of 12.3 (range 1 to 36) years, contributed 74 assessments to this study. The average SELENA SLEDAI was 2.21. The average pre-laboratory PGA was 0.45 and the average post-laboratory PGA was 0.55 (*P *= 0.02 paired Student's *t *test). Twenty-six pre-laboratory PGAs were evaluated as 0, 23 remained at 0 after receipt of laboratory examinations, and three increased secondary to new lupus serologic activity. The pre-laboratory and post-laboratory PGAs were equal in 17 of 53 encounters with disease activity (that is, PGA > 0). The average pre-laboratory PGA of these 53 encounters was 0.67, the average post-laboratory PGA was 0.77 (*P *< 0.05, paired Student's *t *test) and the average SELENA SLEDAI was 3.12 (range 0 to 8).

## Conclusions

In some lupus patients, the PGA determined prior to receipt of laboratory values may be the same as the PGA determined after labs are received. However, in these preliminary data, there was a significant difference between pre-laboratory and post-laboratory PGA. Further studies in a larger patient population are needed to confirm and extend these findings.

